# Carbon catabolite repression correlates with the maintenance of near invariant molecular crowding in proliferating *E. coli* cells

**DOI:** 10.1186/1752-0509-7-138

**Published:** 2013-12-12

**Authors:** Yi Zhou, Alexei Vazquez, Aaron Wise, Tomoko Warita, Katsuhiko Warita, Ziv Bar-Joseph, Zoltán N Oltvai

**Affiliations:** 1 Department of Pathology, University of Pittsburgh, School of Medicine, S701 Scaife Hall, 3550 Terrace Street, Pittsburgh, PA 15213, USA; 2 Department of Radiation Oncology and Center for Systems Biology, Rutgers Cancer Institute of New Jersey, Rutgers, The State University of New Jersey, New Brunswick, NJ 08903, USA; 3 Lane Center for Computational Biology, Carnegie Mellon University, Pittsburgh, PA 15217, USA; 4 Machine Learning Department, Carnegie Mellon University, Pittsburgh, PA 15217, USA

**Keywords:** Metabolic network, Carbon catabolite repression (CCR), Macromolecular crowding (MC), Growth rate

## Abstract

**Background:**

Carbon catabolite repression (CCR) is critical for optimal bacterial growth, and in bacterial (and yeast) cells it leads to their selective consumption of a single substrate from a complex environment. However, the root cause(s) for the development of this regulatory mechanism is unknown. Previously, a flux balance model (FBAwMC) of *Escherichia coli* metabolism that takes into account the crowded intracellular milieu of the bacterial cell correctly predicted selective glucose uptake in a medium containing five different carbon sources, suggesting that CCR may be an adaptive mechanism that ensures optimal bacterial metabolic network activity for growth.

**Results:**

Here, we show that slowly growing *E. coli* cells do not display CCR in a mixed substrate culture and gradual activation of CCR correlates with an increasing rate of *E. coli* cell growth and proliferation. In contrast, CCR mutant cells do not achieve fast growth in mixed substrate culture, and display differences in their cell volume and density compared to wild-type cells. Analyses of transcriptome data from wt *E. coli* cells indicate the expected regulation of substrate uptake and metabolic pathway utilization upon growth rate change. We also find that forced transient increase of intracellular crowding or transient perturbation of CCR delay cell growth, the latter leading to associated cell density-and volume alterations.

**Conclusions:**

CCR is activated at an increased bacterial cell growth rate when it is required for optimal cell growth while intracellular macromolecular density is maintained within a narrow physiological range. In addition to CCR, there are likely to be other regulatory mechanisms of cell metabolism that have evolved to ensure optimal cell growth in the context of the fundamental biophysical constraint imposed by intracellular molecular crowding.

## Background

Carbon catabolite repression (CCR) denotes the phenomenon of selective substrate uptake from complex media by yeast and bacterial cells [1]. Indeed, *E. coli* strains with defective CCR display slower glucose uptake and growth [2-4], suggesting that CCR contributes significantly to their survival and proliferation in ever changing nutrient conditions. CCR is mediated by various mechanisms, including transcriptional repression and protein-protein interaction-mediated inhibition of substrate uptake- and catabolism related proteins [5,6]. However, the fundamental reason(s) for the development of this regulatory mechanism remains poorly understood.

In a previous study, we observed the characteristic CCR in *E. coli* cells that were grown in batch culture in a medium containing an equal mix of five different carbon substrates [7]. In that study we developed a constraint-based modeling framework [8], called flux balance analysis with macromolecular crowding (FBAwMC). This model has successfully predicted the observed sequential substrate uptake kinetics by using a modified form of FBA, which takes into account the total enzyme occupancy limit inside the cell due to the highly crowded nature of the cell’s cytoplasm [7].

Volume exclusion by the presence of macromolecules (macromolecular crowding [MC]), has various effects on biochemical reactions both *in-vitro* and inside the cell [9,10]. *In-vitro* systems show that increased macromolecular concentration attenuates diffusion limited reactions [11,12] but accelerates the transition state-limited reactions [13], accelerates and stabilizes gene-and protein expression [14], promotes correct protein folding by extending association between the unfolded polypeptides with chaperon proteins [15] and prevents protein aggregation [16]. In *E. coli* cells, in which the concentration of total protein and RNA is in the range of 200 ~ 300 g/l [17], increased macromolecular density enhances the self-association of bacterial cell division protein, FtsZ [18], increases PTS (phosphor-transferase system) flux and activities [19], and may promote the reorganization of cell metabolism in rapidly proliferating cells from oxidative phosphorylation (OxPhos) to simultaneous OxPhos and aerobic glycolysis [20]. Moreover, experimental and theoretical studies indicate that normally functioning cells maintain their intracellular macromolecular density within a narrow physiological range [21-26].

In light of these observations, we have hypothesized that CCR is a regulatory mechanism for the maintenance of a near constant intracellular macromolecular density in cells producing biomass at a rapid rate. To test this hypothesis, in this study we characterize the growth of *E. coli* cells both in single substrate-limited and mixed substrate cultures. We find that in mixed substrate cultures *E. coli* cells do not display CCR at slow growth rates and that the gradual activation of CCR correlates with the increasing rate of *E. coli* cell growth and proliferation. We also find that a forced transient increase of intracellular macromolecular crowding (MC) or perturbation of CCR delays cell growth. Moreover, cell density and volume alteration were associated with CCR perturbation. Thus CCR appears to represent an adaptive mechanism that contributes to the maintenance of physiological intracellular macromolecular density in bacterial cells for optimal cell growth.

## Results

### *E. coli* cells display slower substrate uptake and growth rate in single carbon-limited-than in mixed substrate cultures

**Figure 1:**
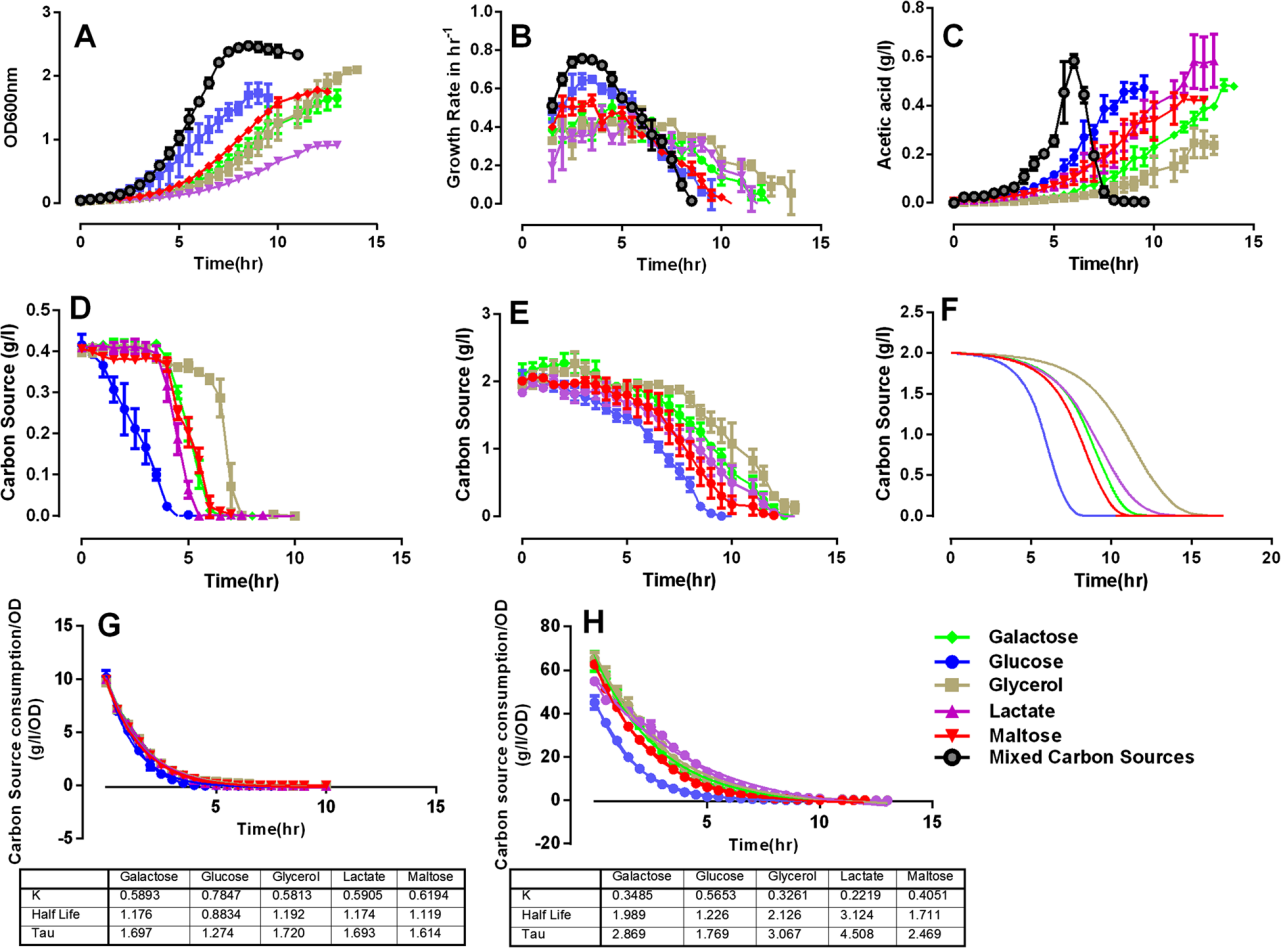
***E. coli *batch cultures in mixed carbon- and single carbon limited media.** Each carbon substrate concentration was at 0.2% in single substrate cultures and 0.04% in the mixed substrate experiment. **A**. Culture growth rates as measured by tracing OD_600_; **B**. Relative cell growth = ln(OD_600t_ – OD_600t-1_); **C**. measured acetic acid secretion rates; **D**, **E**. substrate consumption rates in **D**./ mixed substrate culture and **E**./ single substrate cultures; **F**. the FBAwMC modeling prediction of substrate consumption kinetics in single substrate culture conditions. **G**, **H**, The substrate consumption curves **(D and E)** were normalized to the OD_600nm_ data **(A)** in individual culture conditions (shown by the dotted points). Curve fitting with one phase decay equation was applied to the normalized data in **(G)** mixed substrate culture and **(H)** single substrate cultures (shown in the continuous lines). K is the rate constant and a higher K value indicates a faster substrate uptake. Half-life shows the time points when the normalized substrates concentration decreases to 50%. The data for the black tracings in panels **A**, **B**, and the data for panel **D** are from Ref. [7]).

We previously characterized the culture density-, growth rate- (Figure 1A, B, black lines, respectively) and substrate uptake kinetics (Figure 1D) of *E. coli* cells in mixed substrate culture, and also determined the level of acetate, a well-known metabolic byproduct of rapidly dividing *E. coli* cells, in that culture’s supernatant (Figure 1C, black line) [7]. The carbon source consumption profiles we observed [7] were compatible with the presence of carbon catabolite repression (CCR) in the culture, in which the sole consumption of glucose preceded the concomitant utilization of all other substrates (Figure 1D). To better understand the root cause of the observed substrate uptake patterns, we grew *E. coli* cells separately in the individual components of the mixed culture medium (i.e., in single carbon-limited media), the experiments being terminated upon substrate exhaustion from the growth media or when cells entered the stationary phase.

Compared with the mixed substrate culture, the culture density (Figure 1A) and growth kinetics (Figure 1B) of *E. coli* cells in single carbon-limited media significantly differed, glucose-limited cultures displaying the fastest-and lactate-limited cultures the slowest growth. In terms of their maximal biomass glucose, maltose-, and galactose-limited cultures were comparable and slightly below the glycerol-limited culture (which achieved the highest biomass), while lactate-limited growth resulted in about one half of that level (Figure 1A). As in the mixed culture, all growth rate kinetic curves displayed an initial increase, peaked in the early exponential growth phase, and then decreased (Figure 1B). However, compared to the growth rate seen in the mixed substrate culture the curves were substantially flattened for the galactose-, lactate-, and glycerol-limited cultures and slightly less flattened for the glucose- and maltose-limited cultures (Figure 1B). This behavior correlated with their delayed substrate uptake rate from the culture media (Figure 1E). Acetate (i.e., acetic acid) secretion by *E. coli* cells mirrored these trends, except that in contrast to that seen in the mixed substrate culture (Figure 1C, black tracing) on a population level there was no evidence for acetate reuptake in the single carbon cultures (Figure 1C).

### The FBAwMC model predicts the substrate uptake order of *E. coli* cells both in single carbon-limited- and in mixed substrate cultures

The substrate consumption rates in the five single carbon-limited cultures (Figure 1E) correlate with their apparent substrate uptake order in mixed substrate culture (Figure 1D). In both culture types glucose was utilized first (and at the fastest rate) and glycerol last (and at the slowest rate), while the other three substrates displayed slightly different rank order among the single substrate-limited and mixed substrate cultures.

To quantify the rate and order of substrate consumption kinetics independent of total biomass, we normalized the measured substrate consumption (Figure 1D, E) to the biomass data (Figure 1A). It is evident that the order of intrinsic substrate uptake rate among the five single substrate cultures (Figure 1H) has varied from that seen in the mixed culture medium (Figure 1G). The rate of glucose and maltose uptake rates have displayed consistent consumption ranks in both culture types (first and second, respectively), while the other three substrates displayed different consumption orders. Of note, the consumption rates of all the substrates proved higher in the mixed substrate culture than in the single carbon-limited cultures.

We previously developed a model of *E. coli* metabolism to investigate the origin of the substrate hierarchy consumption in mixed substrate cultures [7]. This model, called flux balance analysis with molecular crowding (FBAwMC) obtains the steady-state metabolic flux distribution that result in the maximum biomass production rate given the available nutrients [7,20]. Here we used FBAwMC to model substrate utilization in the single carbon-limited cultures, finding that its predictions (Figure 1F) are similar to the observed uptake profiles (Figure 1E), while the same model without applying MC as a constraint failed to predict this behavior. The modeling and experimental results thus imply that intracellular macromolecular crowding may relate to the presence of CCR in mixed cell culture.

### *E. coli* cells resist the activation of maltose regulon more in mixed substrate- than in single substrate-limited growth media

The presence of a single substrate in a growth medium is known to exert a repressive effect on the uptake of other substrates (reviewed in [6]); For example, glucose represses the transcription of genes encoding transporters of other non-PTS substrates, as previously reported in [7]. Each of the specific substrates also tends to upregulate a spectrum of genes including its own transporter genes due to substrate induction [27,28]. The sequential substrate uptake observed in mixed substrate growth medium (Figure 1D), however, suggest that it is a result of strict CCR. Although substrate induction observed in single substrate culture tended to repress the expression of other substrates’ transporter genes (Additional file 1: Figure S1), the repression should be more stringent when multiple substrates are present. To test this hypothesis, we examined the promoter activities of operons within the maltose regulon upon their induction in single carbon limited- and mixed substrates cultures using promoter-GFP reporter plasmid-containing *E. coli* cells (Additional file 2: Figure S2 and Table S1). We used a real-time monitoring system, in which the ratios of GFP/OD_600nm_ of cells, with or without maltose regulon induction, were calculated to evaluate promoter activities (Additional file 2: Figure S2).

**Figure 2:**
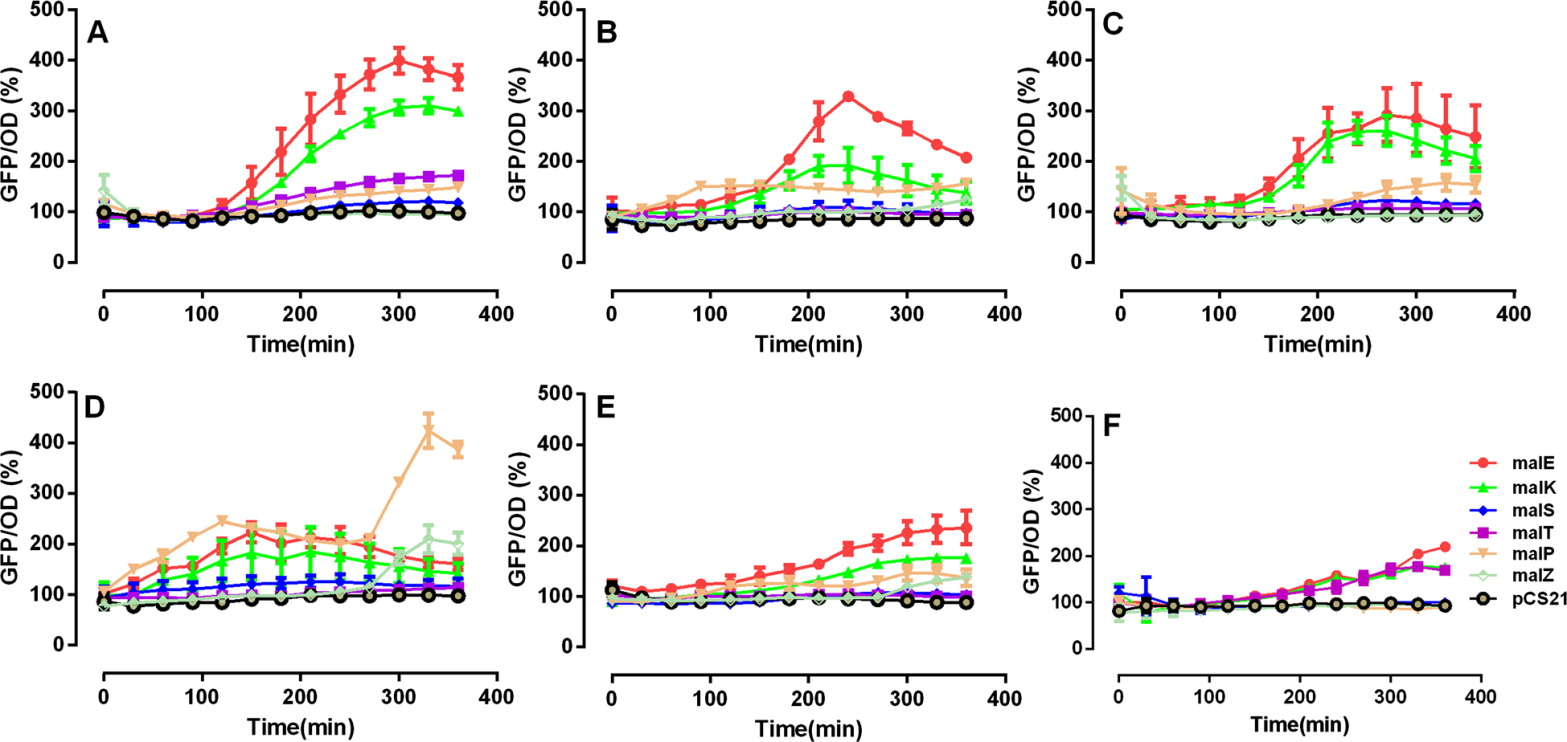
**Maltose regulon responses to its inducers in different growth media.** Response of the maltose regulon genes to induction with 200 μm maltotriose and 4 mM cAMP in **(A)** glucose-, **(B)** galactose-, **(C)** glycerol-, **(D)** lactate-, and **(E)** maltose-limited cultures, and **(F)** mixed substrate medium, as measured by the activity of promoter-GFP constructs.

Selected maltose operon promoters, such as malE and malK, whose gene products are subunits of the maltose transporter, responded strongly to the inducers, maltotriose and cAMP, in glucose-, galactose-, and glycerol-limited cultures (Figure 2A-C) and displayed milder responses in lactate- and maltose-limited cultures (Figure 2D, E). In contrast, delayed and repressed promoter responses were evident in the mixed substrate culture (Figure 2F). Of note, the repressed gene activities in mixed substrate cannot be interpreted as higher basal gene activity because the GFP/OD levels in mixed substrate culture were similar to that in the maltose culture (data not shown). These data indicate the presence of stronger CCR in mixed substrate- than in single substrate-limited growth media.

### The presence and magnitude of CCR is dependent on the biomass production rate of *E. coli* cells in mixed substrate media

To further investigate the potential relationship between CCR and the biomass production (i.e., growth) rate of cells, we next examined substrate consumption behavior in a mixed-substrate *E. coli* culture at various cell growth rates. In continuous-feed chemostat cultures the growth rate of *E. coli* cells can be precisely controlled by changing the culture’s dilution rate. As cells respond to a stepwise increase in the dilution rate of the growth medium, their rate of biomass synthesis and cell division increases [20]. We sampled the chemostat culture 24 hr after increasing the dilution rate (i.e., after the culture has reached a new steady state growth rate [20] and measured the culture density (OD_600nm_), the pH of the growth medium, and the *E. coli* cell volume and cell density.

**Figure 3:**
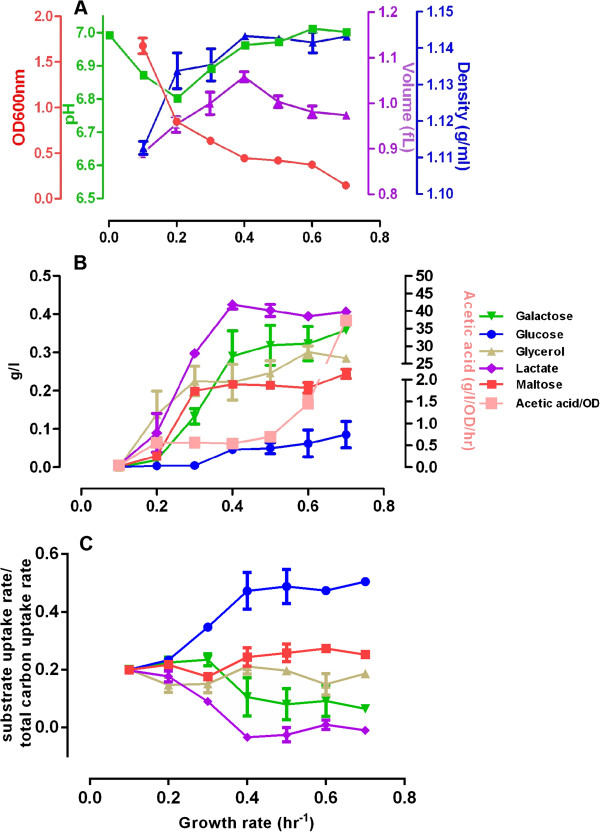
**Continuous-feed, mixed carbon medium chemostat culture of *E. coli *cells.***E. coli* cells were inoculated into the fermenter at an initial OD ~ = 0.04. The flow rate of the continuous-feed culture was adjusted every 24 hr and samples were collected after the cell density has stabilized. **(A)** At the indicated dilution rates samples were tested for culture density (OD_600nm_), pH, cell volume, and cell buoyant density; **(B)** displays the concentration of the indicated substrates of the mixed carbon growth medium at the indicated dilution rates, together with the concentration of the secreted acetate. In **(C)** the consumption ratios, which are calculated as the ratio between individual substrate uptake rate and the total carbon uptake rate, are shown at the indicated dilution rates.

As in glucose-limited culture [20], the cell concentration in the culture medium (i.e., the culture density) decreases with the increased exchange rate of the culture medium (Figure 3A, red curve). Also, the pH of the culture medium decreases slightly to pH ~ 6.8 at 0.2/hr dilution rate but then returns to pH ~ 6.9 and above at growth rates higher than 0.3/hr. This is likely due to the faster dilution rate of the pH 7.0 growth medium (Figure 3A, green curve). From a cell volume of 0.89 fL at the 0.1/hr dilution rate there is an initial increase in the volume of *E. coli* cells with increasing dilution (and cell growth) rate that reaches its peak (1.064 fL) at 0.4/hr, and then decreases and levels off with ~0.98 fL at the highest dilution rate of 0.7/hr (Figure 3A, purple curve). However, the buoyant density of *E. coli* cells displays much less variation. It is lowest (1.11 g/ml) at the lowest dilution/ growth rate at 0.1/hr, and then reaches a medium density (~1.13 g/ml) at growth rates 0.2 and 0.3/hr, respectively. Cell density reaches its highest value of ~1.14 g/ml at 0.4/hr dilution rate that is then maintained till 0.7/hr. These data indicate that while cell volumes change dynamically to match the faster biomass accumulation rate brought on by faster cell growth, cell buoyant density remains remarkably stable and does not increase above a threshold level.

Simultaneously, substrate consumption profiles indicate a clear correlation between the rate of cell proliferation and the presence of CCR (Figure 3B, C, [in Figure 3C, the specific substrate uptake rate is normalized to the total substrate uptake rate]). At the lowest growth rate (at 0.1/hr dilution rate) CCR is absent, as glucose and the other four substrates are utilized fully and simultaneously. At a faster cell growth (0.2/hr) a limited CCR partially inhibiting the consumption of lactate and glycerol is seen (Figure 3B). CCR is more widespread at 0.3/hr when the uptake of galactose and maltose are also partially inhibited (Figure 3B, C). Above this dilution rate there is extensive CCR with predominant (though not exclusive) glucose utilization. Indeed, the utilization of maltose, glycerol and galactose is limited and lactate is not utilized at all at growth rate at and above 0.4/hr (Figure 3B).

The substrate consumption rate compared to total carbon uptake ratio is calculated to describe the percent contribution of each substrates’ uptake to the total carbon uptake along growth rate increase. As shown in Figure 3C, the substrate uptake ratio curves start to diverge after growth rate 0.2/hr, showing that the glucose consumption ratio increases as the growth rate accelerates and it becomes the dominantly utilized substrate. In contrast, as the growth rate increases the substrate consumption ratio of lactate and galactose decreases while that of maltose and glycerol only display a slight change (Figure 3C). This pattern supports the notion that CCR is inactive in slow growing cells but becomes increasingly activated in rapidly growing cells, showing the largest effect at the fastest cell proliferation rate. FBAwMC modeling provides largely similar results and predicts an even more dominant selective glucose consumption at the highest proliferation rates than seen experimentally (Additional file 3: Figure S3).

Of note, at the lowest proliferation rate (0.1/hr) no secreted acetate is present in the growth medium indicating that metabolism takes place exclusively through oxidative phosphorylation (OxPhos). However, with the initial appearance of CCR there is a concomitant presence of secreted acetate in the growth medium, indicating the appearance of partial aerobic glycolysis in the culture (Figure 3B). At very high growth rates (0.6-0.7/hr) there is a second, much larger phase in acetate secretion that may indicate the down-regulation of OxPhos and a dominance of aerobic glycolysis in the culture (Figure 3B). This has been predicted by FBAwMC and is also seen in a glucose-limited continuous-feed chemostat culture [20].

Taken together, these results show that the extent of CCR positively correlates with the *E. coli* cell growth rate, and implies that the activation of CCR in a mixed substrate environment may enable optimal cell growth.

### Transcriptome analysis of mixed-substrate *E. coli* chemostat culture reveals several switches in its metabolic state

To search for transcriptional evidence for the activation of CCR in proliferating *E.* coli cells and to further understand the consequence of growth rate change on *E. coli* cell physiology at the various dilution rates, we collected culture samples for microarray experiments and subsequent transcriptome profiling. We then focused on the relative gene expression levels of the main substrate transport- and catabolism related genes, metabolic enzyme-encoding genes, and genes encoding osmosensor-, anti-stress-, and cell morphology related proteins.

**Figure 4:**
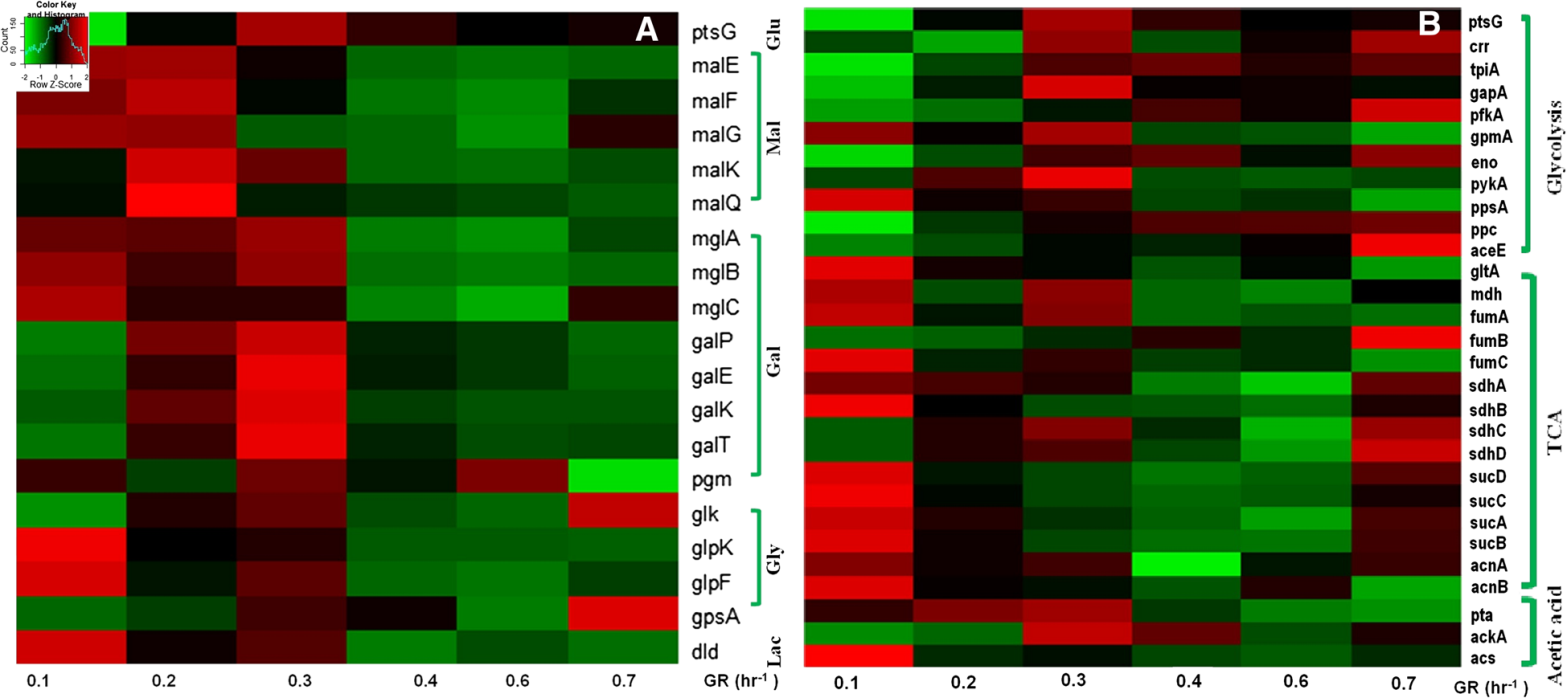
**Gene expression profiles in *E. coli *cells at various growth rates.** Relative gene expression levels of **(A)** the substrate transport and catabolism related genes, **(B)** of genes of the glycolysis pathway, TCA cycle and acetic acid pathway enzymes. Relative gene expression values from the highest (red) to the lowest (green) levels are shown.

Expression of *ptsG*, the gene encoding the glucose transporter PtsG/Crr, is at a high level from the 0.2/hr growth rate with a peak at 0.3/hr (Figure 4A, top row). The low expression level of *ptsG* at 0.1/hr coincides with the high expression levels of the glycerol regulon genes (*glpKF*), the galactose regulon genes (*mglABC*), the maltose regulon genes (*malEFG*) and lactate dehydrogenase gene (*dld*) (Figure 4A). This finding is in agreement with the concomitant utilization of all substrates at this growth rate (Figure 3B, C). The expression level of the transporter genes for all five substrates remained increased at 0.2 and 0.3/hr growth rates indicating active and simultaneous substrate consumption at the slow growing phase. At 0.3/hr there is also an increased expression of the *galEKPT* whose gene product is responsible for the transformation of galactose to intracellular glucose (Figure 4A). However, with further increase in the culture growth rate the expression levels of most transporter genes have decreased sharply, except that of *ptsG*’s, whose expression was still maintained at a medium level from 0.3 ~ 0.7/hr, corresponding to the continued glucose consumption at all growth rates (Figure 3B, C). Of particular interest, the gene encoding the alternative glucose transporter, glucokinase (*glk*), which binds to malT to repress the expression of maltose regulon genes [29], peaks at the highest growth rate, 0.7/hr, when overall glucose uptake rate is at its highest (Figure 3C). These results are consistent with the observed substrate consumption kinetics (Figure 3B, C) and support the notion that CCR is only fully activated at a growth rate above 0.3/hr.

We next examined the correlation between the expression of genes on specific metabolic pathways and the observed growth rate. Most TCA cycle related genes were expressed highly only at the slow growth phase (<0.2/hr) (Figure 4B). In contrast, the expression of glycolysis pathway genes were initially repressed, followed by an initial peak expression at 0.3/hr growth rate and a second peak at the highest growth rate phase (0.7/hr) (Figure 4B). A largely similar transcriptional shift from TCA cycle gene activation to the utilization of glycolysis pathway genes with increasing growth rate was observed previously in a glucose-limited continuous-feed chemostat experiment [20], implying that the selection of the metabolic pathway enzyme activities is more growth rate dependent than substrate dependent.

The activity of the glycolysis pathway is partially reflected by its metabolic byproduct, acetate, whose extracellular concentration is the consequence of a balance of its rate of secretion (via *pta* and *ackA*) and re-uptake (*acs*). We find that acetate production genes (*pta* and *ackA*) are expressed highly between 0.2 ~ 0.4/hr growth rates (Figure 4B). Besides the metabolic switches, osmosensing and cell morphology related genes were also found to be activated differentially along growth rate increase (Additional file 4: Figure S4) indicating their potential involvement in MC optimization of dynamic cell growth.

### The CCR mutant, ΔptsG, displays growth defects and altered cell density in mixed substrate chemostat culture

*E. coli* strains with defective CCR display slower glucose uptake and growth. The ΔptsG mutant with the deletion of the glucose transporter gene encoding EIICB^Glc^ displays repressed glucose consumption with concomitant growth defects in glucose culture [1,3] yet elevates CCR repression when grown in media containing other substrates such as xylose and arabinose [30].

**Figure 5:**
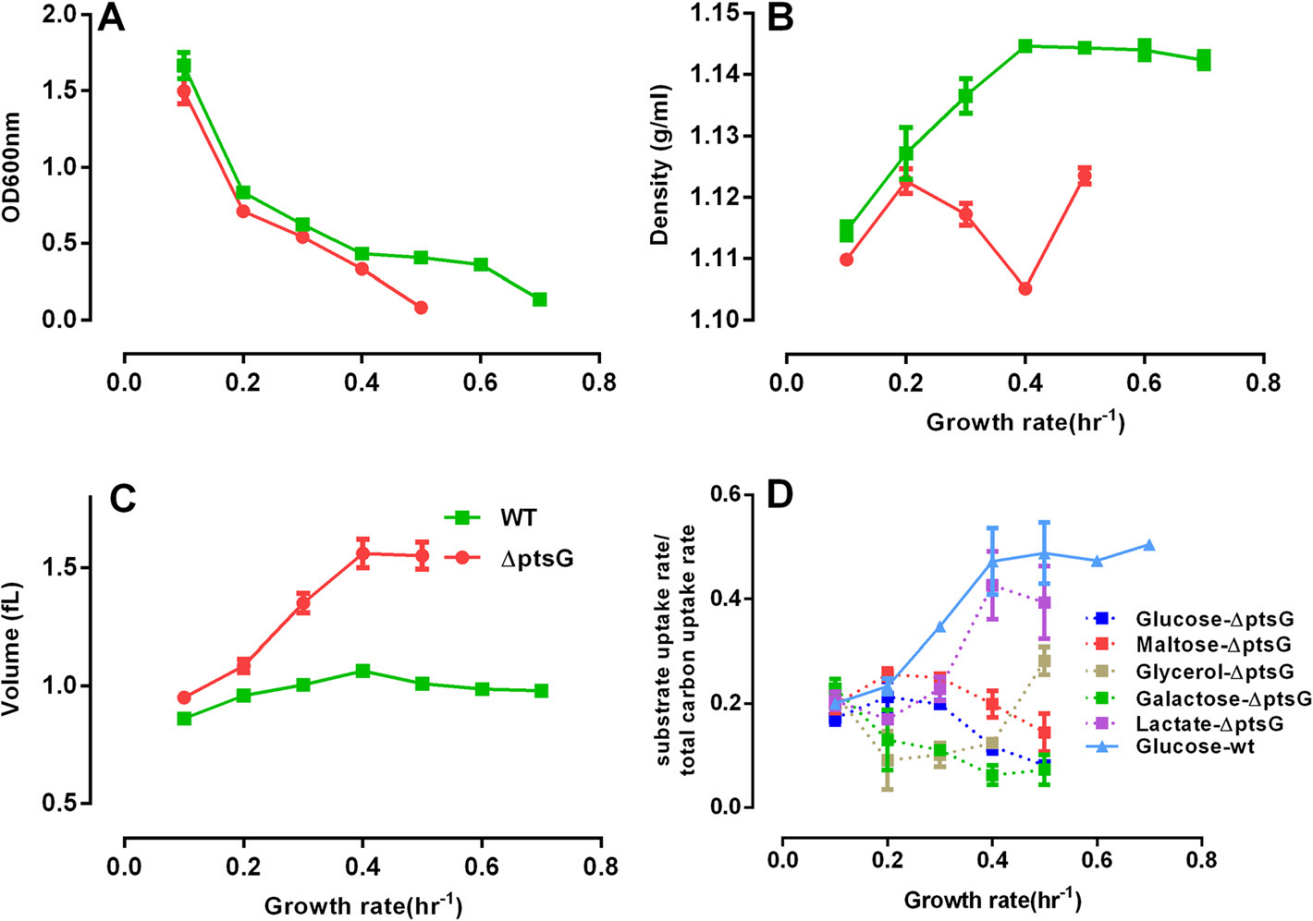
**Continuous-feed, mixed carbon medium chemostat culture of ΔptsG mutant cells.** At the indicated dilution rates samples were tested for **(A)** culture density (OD_600nm_), **(B)** cell buoyant density and **(C)** cell volume comparison; together with the consumption ratio **(D)**, which is calculated as the ratio between individual substrate uptake rate and the total carbon uptake rate and for illustration purpose the glucose consumption ratio of wild type MG1655 cells from Figure 3C is also shown.

When grown in rate-controlled mixed substrate chemostat culture, *E. coli* ΔptsG cells displayed similar cell culture density to wild type cells at growth rate 0.1 to 0.4/hr, but could not reach steady state growth above 0.5/hr (Figure 5A). Interestingly, mutant cells displayed lower buoyant density (Figure 5B) and larger cell volume (Figure 5C) than wild type cells, implying that physiological cell density and volume regulation is intertwined with CCR.

As expected, the measured uptake rates of the five substrates have diverged significantly (Figure 5D) from that seen in the wt *E. coli* culture (Figure 3C). Glucose consumption (through alternative glucose uptake mechanisms) remained very low throughout, while glycerol and lactate displayed the highest uptake rates at the faster growth rates. Interestingly, the consumption of maltose and galactose also remained suppressed, probably because their transporters, such as *galP*, were allocated to transport glucose as the shared transporter, besides the maltose transporter *malEFG*[31]. The altered substrate metabolism phenotype of ΔptsG mutant cells is associated with morphology and intracellular density alteration.

In summary, these data demonstrate that defective CCR results in cell growth defects (with cell volume and density alterations) only at a fast growth phase but not at a slow growth phase, further suggesting that CCR is required for rapid cell growth and cell physiology regulation.

### Transient CCR disruption in mixed substrate *E. coli* culture is associated with cell growth inhibition and cell density alteration

CCR is activated in rapidly growing *E. coli* cells (Figure 3), while in the CCR mutant, *E. coli ΔptsG* cells, MC alteration is associated with growth defects in the fast growth phase (Figure 5A and B). However, CCR-negative mutants always have compromised glucose uptake [3,4] (Figure 5D) and potentially have adapted to a new MC level in the extended period of culture. Therefore, we also wished to examine the cell’s response to transient disruption of CCR. To this end, we induced maltose uptake in a mixed substrate culture with the addition of cAMP (4 mM) plus maltotriose (200 μM) (Additional file 2: Figure S2) at a proliferation rate when maltose uptake is normally inhibited (Figure 1D).

**Figure 6:**
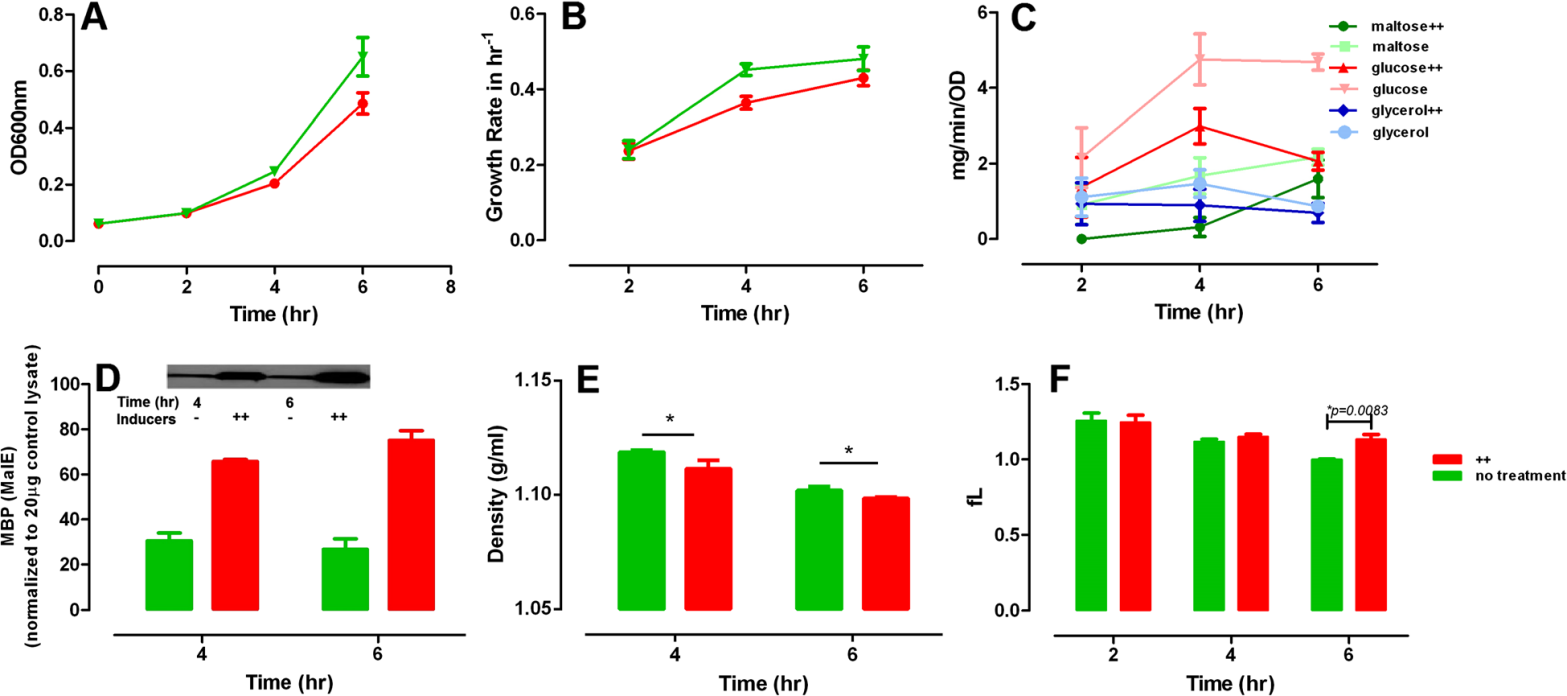
**Cell growth and substrate consumption are inhibited by inducers in batch culture of MG1655 cells in mixed substrate.** Overnight LB cultures of *E.* coli cells were inoculated into 125 ml flask with 15 ml M9 media with 0.2% mixed substrate. The initial OD_600_ was ~0.03 ~ 0.04 and cells were cultured for 6 hr on a shaker with 200 rpm at 37°C. The inducers, 4 mM cAMP and 200 μM maltotriose (++) were added after 15 min culture. Cell growth **(A)** and growth rate **(B)** were sampled every 2 hr and substrate consumption rate of glucose, maltose and glycerol were determined **(C)**. Maltose transporter MBP (MalE) level (the inset showing the immunostaining result) **(D)** and cell density **(E)** were measured at 4th and 6th hr by Ficoll gradient and western blot respectively. The protein expression level in **(D)** was determined with ImageJ. Cell volume **(F)** was measured by Beckman Coulter.

First, we tested if the inducers are sufficient to overcome the CCR-induced repression caused by glucose on the maltose regulon genes. We determined the protein expression level of MalE (or MBP), a subunit of the maltose transporter complex, by western blot analysis after 3.5 hr of induction. We found that MalE expression level was significantly increased upon maltose regulon induction (Figure 6D), indicating that the inducers are able to upregulate maltose transporter expression.

Next, we studied cell growth upon the inducers’ addition. We observed that cAMP plus maltotriose treated cells displayed slightly inhibited cell growth compared to uninduced cells (Figure 6A, B). Simultaneously, the substrate consumption rate of *E. coli* cells displayed decreased uptake of maltose and glucose, with maltose consumption eventually accelerating (Figure 6C). In contrast, the uptake of glycerol was unaffected. We also found a significant increase in cell volume (Figure 6F) and a slight decrease in cell density (Figure 6E) upon induction of the maltose regulon. Thus, upon inappropriate activation of the maltose regulon *E. coli* cells expand their cell volume with concomitant slight delay in cell growth, perhaps in part to accommodate extra proteome mass. Indeed, increase in proteome mass by inducing the expression of an exogenous protein yields a similar growth phenotype (Additional file 5: Figure S5). These data suggest that optimal intracellular MC is constantly sensed and dynamically adjusted along CCR activation.

## Discussion

Carbon catabolite repression (CCR) is an important regulatory mechanism in bacteria that ensures that the cell relies on its preferred substrates to achieve optimal growth [1]. CCR may provide a similar function to the stringent response regulatory mechanism [32] by being sensitive to the growth needs (i.e., to the availability or the lack of nutrients) of the cell. It may act by turning on/off metabolic pathways by facilitating or repressing the necessary molecules of the pathway to ensure optimal cell growth. Indeed, CCR was evident in a mixed substrate batch chemostat *E. coli* culture in the form of sequential substrate consumption (Figure 1D) [7]. This behavior was predicted by a flux balance model that takes into account the crowded intracellular milieu of the cell (FBAwMC) without any prior knowledge of CCR [7]. Nevertheless, the relationship of CCR and intracellular molecular crowding has not been formally examined.

In this study, we show that mixed substrate *E. coli* culture with CCR grows faster and to higher final biomass density than in any of its individual substrates, and that the kinetics of individual substrate uptake is very similar to that predicted by the FBAwMC model (Figure 1). These data suggest that substrate uptake and cell growth relates to macromolecular crowding and that CCR enables optimal cell growth. In turn, we also show that in a mixed substrate culture the extent of CCR’s activation is proportional to the culture’s growth rate while the density of *E. coli* cells remains essentially unchanged (Figure 3). Furthermore, transiently altered CCR in *E. coli* cells decreases their rate of proliferation and growth (Figure 6). Transcriptome analyses (Figure 4) also show that strict transcription level regulation of substrate uptake only appears at higher cell growth rates. Taken together, these data indicate that CCR is a regulatory mechanism that is fully active only during rapid cell growth and may exert its effect on cell function in part by enabling cells to maintain a near constant cytoplasmic density and/or by reducing transporter competition for membrane space [33].

**Figure 7:**
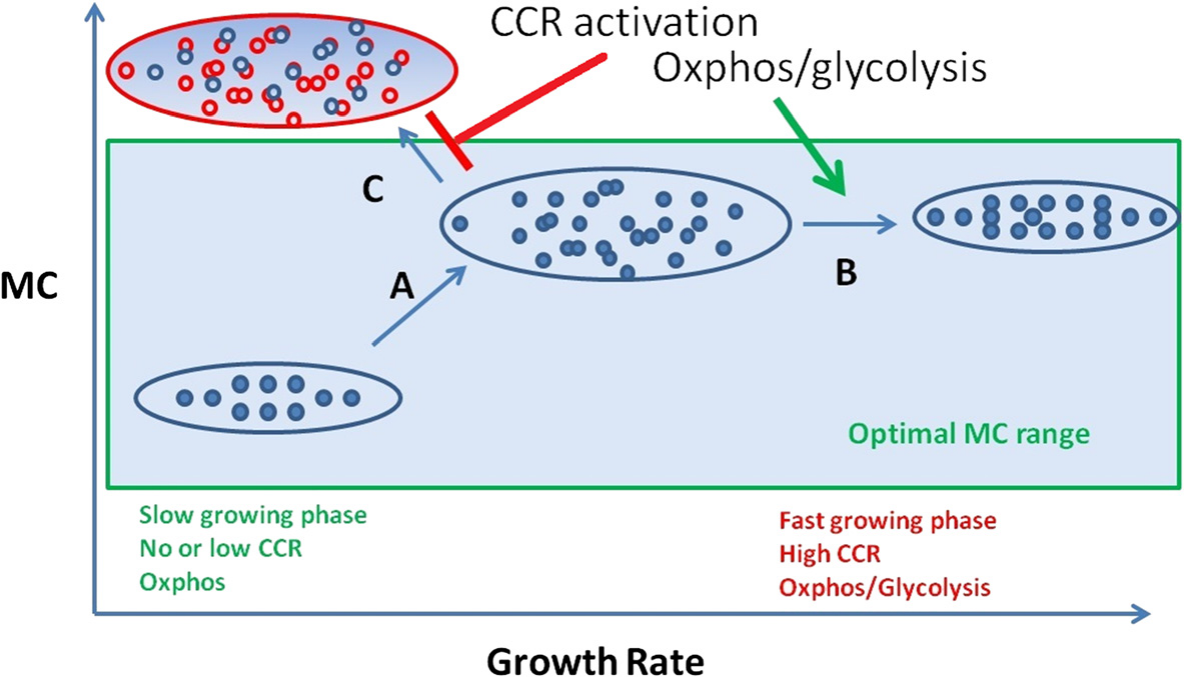
**Model of metabolic adaptations in rapidly proliferating cells.** Slowly growing bacterial cells use oxidative phosphorylation (OxPhos) and CCR is absent. Upon increased growth (transition **A)**, prokaryotes expand cell volume to accommodate the faster biomass accumulation rate. At faster growth rate (transition **B)** prokaryotes also turn on glycolysis and activate CCR in order to maintain cytoplasmic MC within a narrow, optimal range (blue shaded area). In the absence of such accommodation mechanisms cytoplasmic MC would be sub-optimal leading to a reduced growth rate (transition **C)**.

However, CCR may not be the only mechanism that may contribute to the maintenance of intracellular MC by reducing the need for increased total protein (enzyme) content of the cell at rapid growth. Indeed, we have shown previously that with increasing cell growth there is a switch from oxidative phosphorylation (OxPhos) to a “mixed OxPhos with glycolysis” mode both in *E. coli*[20] and mammalian cells [34] that can be interpreted by the same principle. Temporal separation of metabolic activities (i.e., metabolic oscillations) [35-37] may also contribute to the maintenance of optimal intracellular MC. Thus, at slow growth biomass synthesis rate is low and cell metabolism operates in a “substrate limited” mode, in which multiple substrate catabolism pathways are used through OxPhos to maximize substrate uptake and metabolic yield (Figure 7). Yet, when cells are growing rapidly the biomass production rate is significantly higher, which with the same metabolic regime would require higher catalytic proteome content with subsequent increase in MC. To counter this need and to maintain a near constant MC level, bacterial cells initially increase their cell volume to accommodate the extra biomass production and, simultaneously, cell metabolism is reorganized in part to maintain a physiologically optimal MC.

## Conclusions

MC is a biophysical constraint and cell metabolism has to adapt to this fundamental constraint. In rapidly growing cells, CCR and/or the switch to “mixed OxPhos with glycolysis” mode is activated to supply ATP production and substrate catabolism with less MC variation (Figure 7). Additional regulatory mechanisms of cell metabolism have likely developed in response to the need for maintaining intracellular MC at an optimal level. Uncovering the genes, proteins and pathways involved in the detection and regulation of MC is an important step for the understanding of the system-level regulation of cell metabolism.

## Methods

### Bacterial strains and growth conditions

We used the wild-type *E. coli* MG1655 strain and CCR mutant, *ΔptsG* (obtained from the Keio collection [38], Thermo Scientific), throughout the study. Before inoculation, *E. coli* cells were routinely cultured with agitation at 200 rpm, 37°C in Luria-Bertani broth (Teknova) for 48 hr from a frozen glycerol stock, or 24 hr from an LB-agar plate. After the pre-culture in LB, cells were inoculated into a 125 ml flask with 55 ml 1× M9-minimal salts medium (Sigma) with various carbon substrates. The 1 × M9-minimal salts media was supplemented with MgSO_4_ and CaCl_2_ to final concentrations of 2 mM and 0.1 mM, respectively. In single substrate culture experiments, the concentration of substrates was 0.2% w/vol. In mixed substrate culture experiments, five substrates (glucose, glycerol, galactose, lactate and maltose) were added such that each of the substrates had a final concentration of 0.04% w/vol. The cells were cultured in flasks for 6 hr continuously at 260 rpm, 37°C. For fermenter batch culture, cells were inoculated after LB-preculture. In continuous-feed chemo-stat cultures, cells were grown in a continuous growth mode at 7 different dilution rates (0.1, 0.2, 0.3, 0.4, 0.5, 0.6, 0.7/hr) in a Labfors bioreactor (Infors, Switzerland). The growth of the bacterial culture was regularly monitored at OD_600nm_ to document steady state. For all the experiments, the initial OD of the cells was at OD_600nm_ ~ 0.035.

### GFP reporter system of maltose regulon genes

The GFP reporter system was constructed as described in [39]. The promoter regions and binding sites of maltose regulon genes (*malEKSTPZ*) that participate in maltose biosynthesis in *E. coli* MG1655 were PCR amplified using *E. coli* genomic DNA from the sequenced *E. coli* genome (http://ecocyc.org). The functions of the genes were introduced in Additional file 2: Table S1. The primers were designed using Invitrogen® custom design software to amplify the sequences that contain the promoter region with CRP and all other transcription factor binding sites for the gene of interest, with an extension of ~100 bp before the first TF binding site in each case. Both the forward and reverse primers were designed with either XhoI or BamHI restriction site tails.

Plasmids that showed the presence of an insert of correct size on gel were extracted, purified and sequenced. The inserts that showed 100% match were further selected for second round of subcloning into a site upstream of a promoterless low-copy reporter plasmid, pCS21 (obtained from Dr. M. Surette, University of Calgary, AB, Canada) in JM109. The selected gene inserts were then grown in large batches of 150 ml LB-Amp cultures, followed by plasmid purification. These plasmids were again digested by the suitable restriction enzyme followed by gel extraction and purification of the insert to confirm the correct insertion. Then the vectors were transferred into MG1655 cells.

### Plasmids

High-copy reporter plasmids (pET28a), in which an inserted promoter can control the expression of the UCH gene were obtained from Dr. Hao Liu, University of Pittsburgh.

### Batch culture experiments with inducer induction

Before inoculation into 24-well plate culture, *E. coli* cells were routinely cultured with agitation at 200 rpm, 37°C in Luria-Bertani broth (Teknova) overnight from a glycerol stock, or from an LB-agar plate. In flask culture, LB overnight cultured cells were inoculated as introduced above. The cells were cultured in the flasks for 6 hr continuously at 200 rpm, 37°C. Inducers (4 mM cAMP and 200 μM maltotriose) were added after 15mins culture and cell culture was monitored for 6 hr. Cell culture media was sampled to determine OD_600nm_ and substrate consumption.

### Cell growth and growth rate calculation

The cell growth data were collected at 30 min intervals and 150 μl aliquot of the cell cultures were measured by a photometer (Eppendorf) at OD_600nm_. The growth rate (GR) was calculated as:

$$\left(\frac{{}^{\mathrm{ODt}}{/}_{\mathrm{OD}\left(t-1\right)}}{\mathrm{\Delta t}}\right)$$

Where GR denotes the growth rate at t (hr^-1^), OD_t_: the OD_600nm_ measured at t, and Δt: the sampling interval (hr).

### Cell density measurement with Ficoll gradient

The Ficoll step gradient was prepared by dissolving the Ficoll 400 powder (GE Healthcare) in 1 × PBS to achieve ~ 60%w/v concentration. The density of the solution was measured with a Densito 30PX densitometer (Mettler Toledo). The most condensed Ficoll solution prepared for the experiments was 1.19 g/ml (ρ_0_). The lower density solutions (ρ_1~_ ρ_7_) were prepared from ρ_0_ solution by diluting the dissolved Ficoll solution with PBS. The densities of the gradient used for the cell separation were, ρ_0_- 1.19 g/ml, ρ_1_- 1.18 g/ml, ρ_2_- 1.16 g/ml, ρ_3_- 1.14 g/ml, ρ_4_- 1.12 g/ml, ρ_5_- 1.10 g/ml, ρ_6_- 1.08 g/ml, ρ_7_- 1.06 g/ml. 0.5 ml of each individual gradient solution were gently layered into a 4.5 ml centrifuge tube (Beckman, 344062) from the heaviest to the lightest solution, as previously described [40]. The interface of each two layers was marked on the tube before centrifugation.

In the Ficoll gradient experiment, 10 ml of the cells were centrifuged at 4,500 g × 15 min in Beckman Coulter Allegra 15R at 4°C. The supernatant was removed until only ~0.1 ml culture media was left in the tube. 0.1 ml PBS was added into each tube and the cells were resuspended. The cells were then transferred into the 4.5 ml tube with the 8 layers of Ficoll gradient. More PBS solution was added into the 4.5 ml tube to fill the remaining space to prevent cracking during the high speed centrifugation. The prepared tubes were loaded into a SW60 Ti rotor and subjected to centrifugation at 16,000 g × 1 hr at 4°C in an L8 Ultracentrifuge (Beckman Coulter). The cells distributed into each of the layer that has had the most similar density to the cells. At the end of the run, 0.5 ml of each layer was carefully transferred into a 70 μl UV-Cuvette (BRAND) and the solution was mixed through pipetting. The OD was measured at 600 nm with a Photometer (Eppendof) and the background of the same Ficoll gradient solution was subtracted from the readout. The cell density distribution (CDD) was calculated as:

$$\mathrm{CDD\rho i}=\frac{\mathrm{OD\rho i}}{\sum_{i=0}^{7}\mathrm{OD\rho i}}$$

### Cell volume measurement

After adjusting the dilution rate of the feeding medium, cells in chemostat culture were sampled several times a day, until after the growth rate had been stabilized. Cell culture media was vortexed for 5 seconds then 20 μl cell suspension for each sample was measured by diluting in 20 ml suspension solution (Beckman Coulter) then loaded into Multisizer 3 (Beckman Coulter) for cell volume measurements. Each sample was measured twice.

### Western blotting

For each sample, 20 μg of protein lysates were mixed with 1 × sample buffer, boiled for 5 min., and then loaded onto 15% SDS-PAGE, 100 V for 2 hr. The gel was transferred on a nitrocellulose membrane (Bio-Rad) at 25 V for 2 hr. The membrane was blotted with a mouse monoclonal MalE antibody (NEB) (1: 3000) overnight at 4°C. The membrane was then washed 3 × 5 min with TBST buffer. The secondary HRP-anti mouse (1:10,000) antibody was used to probe the membrane for 1 hr at RT. The membrane was washed 3 times with TBST then processed for chemiluminescence development.

### Substrate concentration measurement

The sampled cell culture (1.5 ml) at different time points was subjected to centrifugation at 4,500 × *g* for 10 min at 4°C. The supernatant was transferred to a 1.5 ml tube and incubated at 80°C for 15 min to deactivate the enzymes. Substrate concentrations were determined according to the manufacturer’s protocol (R-Biopharm, Germany).

### Testing maltose regulon promoter activities

For measuring the promoter activities of operons within the maltose regulon upon their induction in single carbon limited- and mixed substrates cultures, we first created promoter-GFP reporter plasmid-containing *E. coli* cells, as shown in Additional file 2. The inducers, cAMP and maltotriose activate the promoters of the maltose regulon genes to synthesize GFP. This is the indirect measurement of the activities of the maltose regulon genes. We used a real-time monitoring system, in which the ratio of GFP/ OD_600nm_ between cells with or without maltose regulon induction were calculated to evaluate promoter activities.

### Microarray experiments

For the chemostat culture experiments, cells were sampled at every 24 hrs after the dilution rate was adjusted. The whole cell culture volume (45 ml) was mixed with 5 ml of ice-cold stop-solution (5% water-saturated phenol in absolute ethanol), and a cell pellet was obtained by centrifugation at 4,500 × *g* for 10 min at 4°C, followed by flash freezing of pellets with liquid nitrogen. The pellets were stored at −80°C until further use. RNA was isolated from the frozen cell pellets by using Epicenter’s Masterpure RNA isolation kit (using the manufacturer’s product manual). The samples were also treated with DNase for 1 hr at 37°C to remove DNA contamination in the RNA samples. Ten micrograms of all RNA samples were processed for transcriptome analysis using *E. coli* Affy-metrix microarray chips by the Microarray Resource Centre, Department of Genetics and Genomics at Boston University School of Medicine http://www.bumc.bu.edu/microarray/. Microarray data were processed in R using the ‘affy’ package. Background correction was done with MAS5, which is Affymetrix’s recommended procedure. For Figure 3 normalization of gene expression values was performed first with qspline; cross-array normalization was then done using R’s version of dchip [41]. Relative gene expression values from the highest (red) to the lowest (green) are shown.

### FBAwMC model

The simulations of mixed and single substrate cultures were performed using the FBAwMC model reported in Ref. [20] and described in Additional file 3.

### Statistics

Values are expressed as the mean ± SD. Data are plotted and regressed with Graphpad Prism5 (Graphpad Scientific). Intergroup differences were assessed by using the t-test.

### Availability of supporting data

Microarray data have been deposited in Gene Expression Omnibus (GEO accession number: GSE51581).

## Abbreviations

CCR: Carbon catabolite repression; FBAwMC: Flux balance model with macromolecular crowding; MC: Macromolecular crowding; OxPhos: Oxidative phosphorylation; PTS: Phosphor-transferase system; UCHL1: Ubiquitin carboxy-terminal hydrolase-L1.

## Competing interest

The authors declare that no competing financial and non-financial interests exist.

## Authors’ contributions

YZ and ZNO designed the experiments. YZ, TW and KW performed the experiments. AV and AW performed the modeling and microarray data analysis, respectively. YZ, AV, ZBJ and ZNO wrote the manuscript. All authors read and approved the final manuscript.

## Supplementary Material

Additional file 1: Figure S1 Gene expression profiles of substrate catabolism related\transporter genes. Click here for file

Additional file 2: Figure S2 The GFP-reporter system for mal-regulon promoter activity study. **Table S1.** Functions and properties of select genes of the MAL-regulon. Click here for file

Additional file 3: Figure S3 a) Carbon source uptake rates normalized to the total carbon source uptake (%) as a function of the proliferation rate. *U* represents the simulated maximum uptake capacity of each carbon source. b) Left hand side of the molecular crowding constraint (Equation 3) as a function of the proliferation rate. Click here for file

Additional file 4: Figure S4 Gene expression profiles in *E. coli* cells at various growth rates. Click here for file

Additional file 5: Figure S5 Transient protein expression induced MC increase and growth inhibition. Click here for file
