# Supplementary material for: Carbon catabolite repression correlates with the maintenance of near invariant molecular crowding in proliferating E. coli cells
Source: BMC Syst Biol. 2013 Dec 12;7:138. doi: 10.1186/1752-0509-7-138 (PMC3924228; doi:10.1186/1752-0509-7-138)
Supplement: Additional file 1: Figure S1 — Gene expression profiles of substrate catabolism related\transporter genes. [file 1752-0509-7-138-S1.docx]

**Additional file 1**


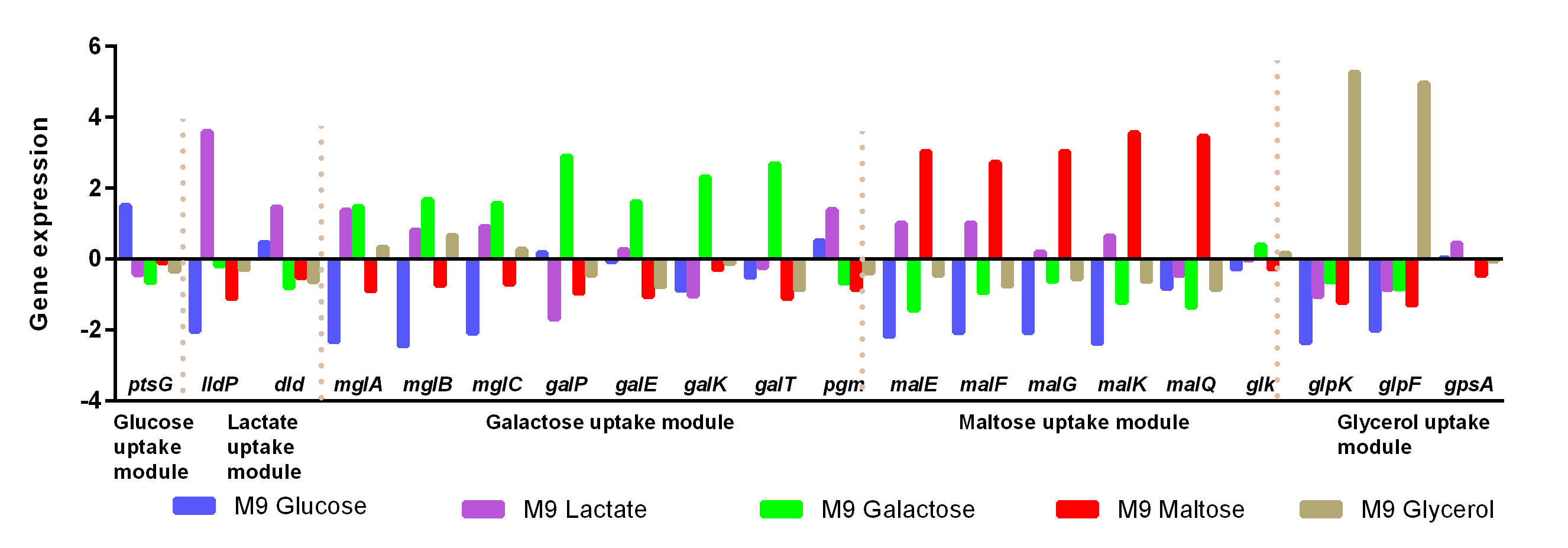


**Figure S1**  **Gene expression profiles of substrate catabolism related transporter genes**

The panel displays the relative mRNA expression of the indicated substrate transporter genes in glucose limited-(blue bar), lactate-limited (purple bar), galactose-limited (green bar), maltose-limited (red bar), and glycerol-limited (grey bar) or mixed substrate media batch cultures at their early exponential growth phase (OD_600nm_=0.2) (data is from Ref [7], main text). It is evident that substrate transport- or catabolism related genes of the unavailable or secondary substrates were repressed in single carbon-limited substrate media, albeit to somewhat differ­ent degrees.
