# Supplementary material for: Carbon catabolite repression correlates with the maintenance of near invariant molecular crowding in proliferating E. coli cells
Source: BMC Syst Biol. 2013 Dec 12;7:138. doi: 10.1186/1752-0509-7-138 (PMC3924228; doi:10.1186/1752-0509-7-138)
Supplement: Additional file 2: Figure S2 — The GFP-reporter system for mal-regulon promoter activity study. Table S1. Functions and properties of select genes of the MAL-regulon. [file 1752-0509-7-138-S2.docx]

**Additional file 2**

**maltotriose**

**cAMP**

***crp***

***malT***

***malS***

***malEFG***

***malK_lamB***

***_malM***

***malPQ***

***malZ***

***malI***

**malXY**

**A**

**A**

***malEKSTPZ***

**MG1655**

**Quantify OD600NM_600nm_ and GFP with fluorescence plate reader**

**Transformation**

**Only sample wells are added with 4mM cAMP and 200μM maltotriose**

pCS21

**In M9 solution with carbon source and 30μg/ml kanamycin**

**GFP**

**B**

**Figure S2 The GFP-reporter system for mal-regulon promoter activity study**

**A.** In the maltose regulon, upon the activation of the transcription factors, Crp and MalT with their regulatory ligands (cAMP and maltotriose, respectively), the transcription of their downstream genes will be activated (green arrows) including the transporter genes malEFG and malK; **B.** Constructing the reporter strains by integrating the promoter sequences of malEKSTPZ to the upstream of the GFP sequence in vector pCS21. The 100 µl O/N cell cultures were added into 1ml M9 solution containing wells. The cells were then cultured for 6 hr at 37°C, 200rpm, and the gene’s activity was recorded as the GFP signal collected from the 24 well-plate at every 15 min or 30 min base. All the data represents the average results of 3~4 separate experiments.

**Table S1: Functions and properties of select genes of the MAL-regulon**

| *Gene* | *Function(s)* |
| --- | --- |
| *malE* | MalKFGE is a maltose transport system that is a member of the ATP-Binding Cassette (ABC) Superfamily of transporters. MalE is the periplasmic maltose-binding protein |
| *malK* | ATP-binding component of transport system for maltose. MalK inhibits the activation of MalT by competing with the binding of the inducer maltotriose to MalT |
| *malS* | Gene encoding the periplasmic α-amylase. The periplasmic alpha-amylase degrades maltooligosaccharides with chain lengths longer than 6 glucose units, which can then be transported through the cytoplasmic membrane. The enzyme hydrolyzes internal 1,4-glucosidic linkages. The enzyme is thought to contain Ca^++^ binding sites |
| *malT* | Positive regulator of MAL-regulon. This regulator participates in controlling several genes involved in maltose utilization. It binds maltotriose and ATP. It activates the transcription of the *malS* gene and the *malEFG, malK-lamB-malM*, and *malPQ* operons |
| *malI* | MalI belongs to the GalR/LacI family of the transcriptional regulators. It is a negative DNA-binding transcriptional regulator member of maltose regulon. *malI* regulates the expression of *malXY* adjacent and divergently operon. It binds maltose as an inducer |
| *malX* | MalX, the maltose-glucose PTS permease, belongs to the functional superfamily of the phosphoenolpyruvate (PEP)-dependent, sugar transporting phosphotransferase system (PTS). The PTS transports and simultaneously phosphorylates its sugar substrates in a process called group translocation. MalX presumably takes up exogenous sugar, releasing the phosphate ester into the cell cytoplasm in preparation for metabolism. The MalX (Enzyme IICB^Mal^) use glucose and maltose as substrate |
| *malZ* | Maltodextrin glucosidase is the product of the *malZ* gene. This enzyme catalyzes the degradation of short malto-oligosaccharides, ranging from maltotriose to maltoheptaose. The enzyme also play a role in regulating the intracellular level of maltotriose |
| *malP* | Codes for enzyme maltodextrin phosphorylase. The maltodextrin phosphorylase has a high affinity for short, linear alpha-1,4 linked oligoglucosides. The shortest maltodextrin which can be readily phosphorylyzed is maltopentaose. The enzyme is dependent on pyridoxal phosphate for activity |
