# Supplementary material for: Carbon catabolite repression correlates with the maintenance of near invariant molecular crowding in proliferating E. coli cells
Source: BMC Syst Biol. 2013 Dec 12;7:138. doi: 10.1186/1752-0509-7-138 (PMC3924228; doi:10.1186/1752-0509-7-138)
Supplement: Additional file 3: Figure S3 — a) Carbon source uptake rates normalized to the total carbon source uptake (%) as a function of the proliferation rate. U represents the simulated maximum uptake capacity of each carbon source. b) Left hand side of the molecular crowding constraint (Equation 3) as a function of the proliferation rate. [file 1752-0509-7-138-S3.doc]

**Additional file 3**

*Flux Balance analysis with Molecular Crowding*

The Flux Balance analysis with Molecular Crowding (FBAwMC) modeling framework is implemented by solving the following optimization problem [1]. Given a metabolic network with *M* metabolites and *N* reactions, find the optimal flux distribution *f_i_*, *i*=1,…,*N*, that maximizes the growth rate subject to the constraints

(1) $\sum_{i=1}^{N} S_{mi}f_{i}=0$ *m*=1,…,*M* (flux balance constraint)

(2) $f_{i}\leq\nu_{i}$ *i*=1,…,*N* (flux capacity constraints)

(3) $\sum_{i=1}^{N} a_{i}f_{i}\leq1$ (molecular crowding constraint)

where *S_mi_* is the stoichiometric coefficient of metabolite *m* in reaction *i*, *ν_i_* is the maximum capacity of reaction *i*, and *a_i_* is the crowding coefficient associated with reaction ii [1]. The maximum growth rate corresponds to the biomass production rate, where biomass production is an auxiliary reaction containing as substrates the cellular components in their relative concentrations and as product the cell’s biomass. Unless specified, we assume *ν_i_*=∞ for every reaction.

*Crowding coefficients*

We model the crowding coefficients *a_i_* as noise. The reported results were obtained assigning a random value to them from the gamma distribution

$$P(a)=\frac{\beta}{\left\langle a\right\rangle}\left(\frac{\beta}{\left\langle a\right\rangle}a\right)^{\beta-1}\exp\left(-\frac{\beta}{\left\langle a\right\rangle}a\right)$$

where *β*>0 and <*a*> is the average crowding coefficient. There is no particular reason for this choice other than by changing *β* we can explore different scenarios. For instance, for *β*=1 we obtain an exponential distribution, while for *β*>>1 we obtain a distribution that is almost concentrated around *a*=<*a*>. The results in Ref. [1] and here were obtained using *β*=3 and running the simulations 1000 times to test the sensitivity of the results with respect to the specific *a_i_* values. Similar results are obtained using other *P*(*a*) distributions (data not shown).

The average crowding coefficient <*a*> was fitted to obtain the minimum square deviation between the measured and model predicted growth rates [1], resulting in <*a*>=0.0040±0.0005 hour DW/mmol. However, the maximum growth rate on glucose and glycerol are more consistent with <*a*>=0.0031±0.0001 hour DW/mmol and <*a*>=0.0053±0.0001 hour DW/mmol, respectively.

*Batch culture simulations*

To model the temporal order of substrate uptake we specify an initial concentration of glucose, galactose, lactate, maltose-, glycerol, zero acetate concentration, and cell’s dry weight *DW*=0.00675 g. The progression of the dry weight and the external substrate concentrations were obtained from the integration of the differential equations

$$\begin{matrix} \frac{dDW(t)}{dt}=\mu(t)DW(t) \\ \frac{dC_{m}(t)}{dt}=\frac{\mu_{m}}{V}\sum_{i} S_{mi}f_{i}(t) DW(t) \end{matrix}$$

where *m* is restricted to external metabolites, *μ_m_* is the molar mass of metabolite *m* and *V* is the working volume. The maximum growth rate *μ*(*t*) and the fluxes *f_i_*(*t*) are obtained by solving the FBAwMC model for the substrate concentration profile at time *t*. The value of <*a*> is smaller if glucose alone is consumed and larger if glycerol is consumed. Therefore, we solve three FBAwMC problems corresponding to the consumption of glucose alone (<*a*>=0.0031 hour DW/mmol), consumption of all substrates except glycerol (<*a*>=0.004 hour DW/mmol) and consumption of all substrates (<*a*>=0.0053 hour DW/mmol), and selected the condition resulting in the maximum growth rate.

*Chemostat simulations*

To model the chemostat culture we simulated a condition where the concentration of the different carbon sources in the feeding media is such that the uptake capacity of each carbon source is the same. In this case *ν_i_*=*U* for each uptake reaction associated with the carbon sources present in the media. By increasing *U* we can simulate an increased concentration of the carbon sources in the media, which in turn results in an increased growth rate. For each value of *U*, the maximum growth rate *μ*(*U*) and the optimal fluxes *f_i_*(*U*) are obtained by solving the FBAwMC model (1)-(3). The value of <*a*> is smaller if glucose alone is consumed and larger if glycerol is consumed. Therefore, we solve three FBAwMC problems corresponding to the consumption of glucose alone (<*a*>=0.0031 hour DW/mmol), consumption of all substrates except glycerol (<*a*>=0.004 hour DW/mmol) and consumption of all substrates (<*a*>=0.0053 hour DW/mmol), and selected the condition resulting in the maximum growth rate.


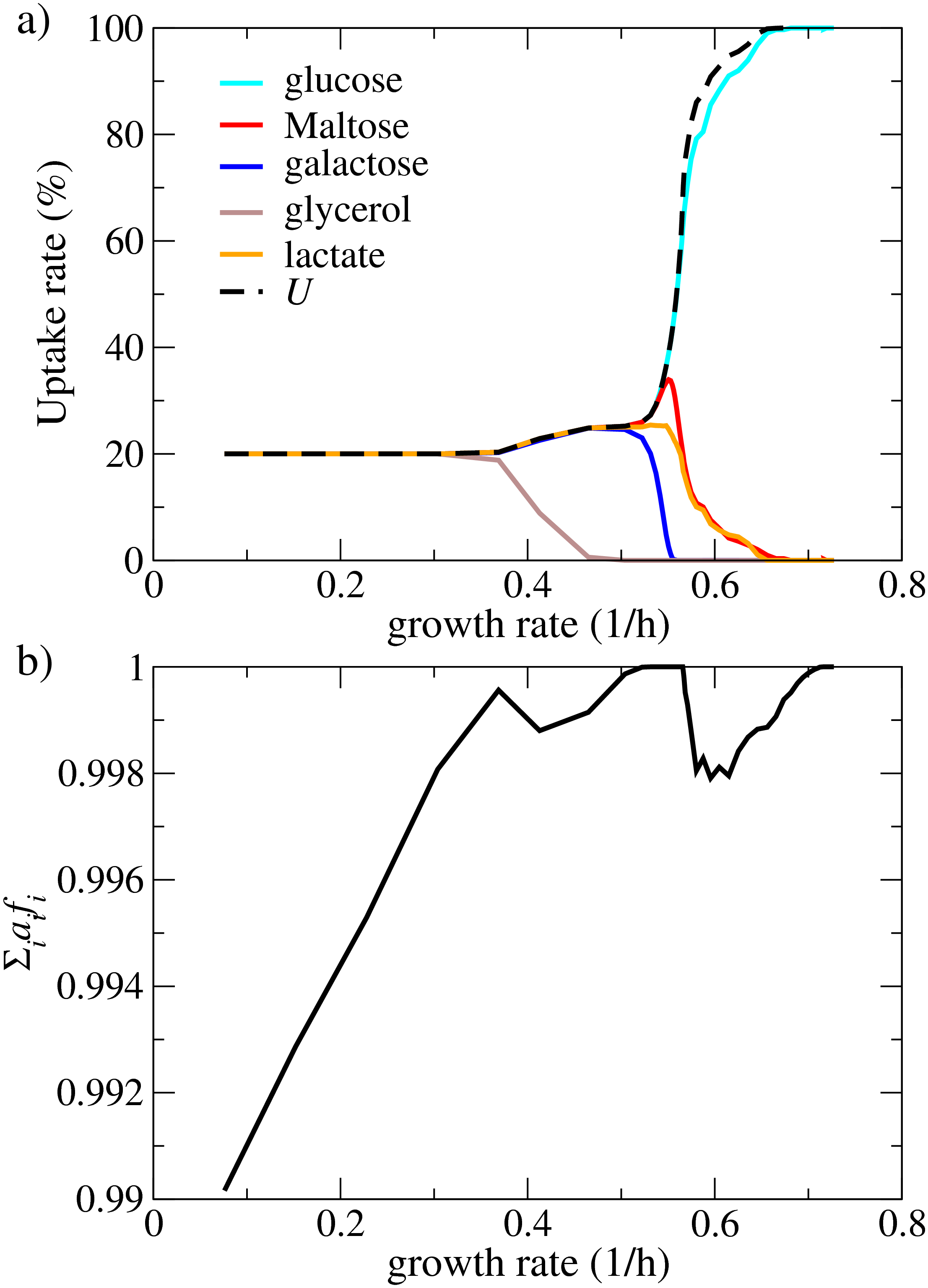


**Figure S3** a) Carbon source uptake rates normalized to the total carbon source uptake (%) as a function of the proliferation rate. *U* represents the simulated maximum uptake capacity of each carbon source. b) Left hand side of the molecular crowding constraint (Equation 3) as a function of the proliferation rate.

**References**

1. Beg QK, Vazquez A, Ernst J, de Menezes MA, Bar-Joseph Z, Barabasi AL, Oltvai ZN: **Intracellular crowding defines the mode and sequence of substrate uptake by Escherichia coli and constrains its metabolic activity**. *Proc Natl Acad Sci U S A* 2007, **104**(31):12663-12668.
