# Supplementary material for: Carbon catabolite repression correlates with the maintenance of near invariant molecular crowding in proliferating E. coli cells
Source: BMC Syst Biol. 2013 Dec 12;7:138. doi: 10.1186/1752-0509-7-138 (PMC3924228; doi:10.1186/1752-0509-7-138)
Supplement: Additional file 4: Figure S4 — Gene expression profiles in E. coli cells at various growth rates. [file 1752-0509-7-138-S4.docx]

**Additional file 4**

**Osmosensor and stress response gene expression in continuous-feed chemostat culture**

With increasing growth rate, we observed cell volume and minimal cell density adjustments (Fig. 4A, main text). As osmosensors are known to be associated with cell volume and density alteration in *E. coli* cells, we therefore also examined the osmosensor genes (*osmYCBE*), stress genes (*rpoSE*) and cell volume related genes (*MreBCD*). We find that osmosensor genes *osmYCBE* are highly expressed at slow growing phases (0.1~0.3/hr) but they are downregulated in the higher growth phase (0.4~0.7/hr) (Fig. S4). This implies that osmosensors may be activated to cooperate with multiple transporter gene activation in the slow growing phase as potential cofactors of cell homeostasis. As found previously, the hunger stress genes, *rpoSE*, are also expressed highly at low growth rates but not at high growth rates (Fig. S4). The results imply that at high culture density multiple substrate consumption activate the hunger stress genes, while the stress level is lowered by growth rate increase with concomitant CCR activation. It is possible that the activation of CCR represses the unnecessary secondary transporter genes’ transcription to lower the demand for cell resource allocation and intracellular volume that result from protein synthesis. Concomitantly, cell morphology genes, *mreBCD* were also gradually upregulated with increasing growth rate, and peaked sharply at the highest dilution rate (0.7/hr). In light of the observed cell volume changes (Fig. 4*A*, main text), it suggests that active cell volume adjustment is coupled with higher proliferation and growth rate.

|  |
| --- |

**Figure S4 Gene expression profiles in *E. coli* cells at various growth rates**

Relative gene expression levels of genes encoding osmosensors, anti-stress and cell morphology related proteins. Relative gene expression values from the highest (red) to the lowest (green) levels are shown.
