# Supplementary material for: Carbon catabolite repression correlates with the maintenance of near invariant molecular crowding in proliferating E. coli cells
Source: BMC Syst Biol. 2013 Dec 12;7:138. doi: 10.1186/1752-0509-7-138 (PMC3924228; doi:10.1186/1752-0509-7-138)
Supplement: Additional file 5: Figure S5 — Transient protein expression induced MC increase and growth inhibition. [file 1752-0509-7-138-S5.docx]

**Additional file 5**

**Transient protein overexpression induces cell density increase and cell growth retardation**

If CCR is indeed an adaptation mechanism that is activated to maintain near constant intracel­lular macromolecular crowding in prolife­rating cells, it is expected that the forced increase of cell density would interfere with optimal cell growth. Indeed, previous studies have shown that constitutive- or inducible exogen­ous protein overexpression attenuates cell growth [[1](#_ENREF_1), [2](#_ENREF_2)]. To confirm these find­ings, we therefore aimed to alter *E. coli* cell den­sity by the transient over-expression of an exogen­ous protein. Specifically, we have used an IPTG-inducible plasmid encoding the rat ubiqui­tin carboxy-terminal hydrolase-L1 (UCHL1) protein. UCHL1 is a small (~25kD) but abundant protein that accounts for 1~2% of soluble cytoplasmic proteins in neuronal cells [[3](#_ENREF_3)]. Therefore, it is not expected to interfere with the intrinsic cell signaling pathways in *E. coli* cells or to cause toxicity.

After 3.5 hr of IPTG induction the expression of UCHL1 was clearly upregulated (see the inset with the western blot result in Fig. *S5A*). No significant cell volume expansion was observed with tran­sient cytoplasmic UCHL1 expression (data not shown); However, cell buoyant density has slightly increased by 3.5 hr of UCHL1 expression (Fig. *S5A*) with simultaneous slight (though statistically not significant) decrease in the density and growth rate of the culture (Fig. *S5B*). Thus a slight negative correlation between exogenous protein expression-induced cell den­sity increase and slightly reduced cell growth rate is evident.


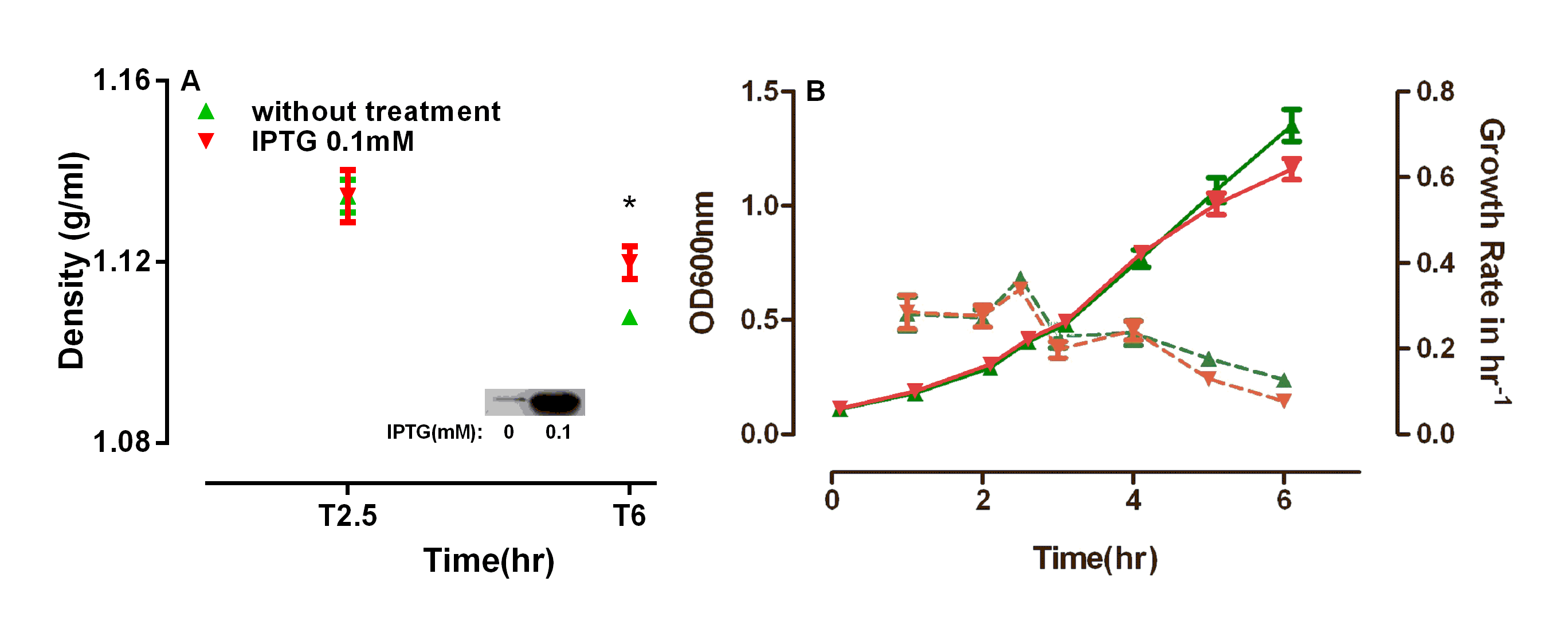


**Figure S5 Transient protein expression induced MC increase and growth inhibition**

(A) IPTG was added to the cell culture at the t=2.5 hr (T2.5). Cell densities were measured at this time point and at 3.5 hr (T6) with/without 0.5mM IPTG induction (see the Western blot insert for comparative UCHL1 protein expression). The asterisk in (A) indicates significant difference in cell density; (B). Culture density (OD_600nm_) (solid line) and calculated growth rate (dotted line) of UCH vector containing *E. coli* cells with (red triangle) or without (green triangle) IPTG induction.

**References**

1. Hoffmann F, Weber J, Rinas U: **Metabolic adaptation of Escherichia coli during temperature-induced recombinant protein production: 1. Readjustment of metabolic enzyme synthesis**. *Biotechnology and Bioengineering* 2002, **80**(3):313-319.

2. Hoffmann F, Rinas U: **On-line estimation of the metabolic burden resulting from the synthesis of plasmid-encoded and heat-shock proteins by monitoring respiratory energy generation**. *Biotechnology and Bioengineering* 2001, **76**(4):333-340.

3. Day INM, Thompson RJ: **UCHL1 (PGP 9.5): Neuronal biomarker and ubiquitin system protein**. *Progress in Neurobiology* 2010, **90**(3):327-362.
